# Mitochondrial β‐carbonic anhydrase is a conserved metabolic rheostat for branched‐chain amino acid catabolism and metabolic flexibility

**DOI:** 10.1111/nph.71056

**Published:** 2026-03-15

**Authors:** Naveen Sharma, Thomas D. Sharkey, Federica Brandizzi

**Affiliations:** ^1^ MSU‐DOE Plant Research Laboratory, Michigan State University East Lansing MI 48824 USA; ^2^ Plant Resilience Institute, Michigan State University East Lansing MI 48824 USA; ^3^ Department of Biochemistry and Molecular Biology Michigan State University East Lansing MI 48824 USA; ^4^ Department of Plant Biology Michigan State University East Lansing MI 48824 USA; ^5^ Great Lakes Bioenergy Research Center Michigan State University East Lansing MI 48824 USA

**Keywords:** *Arabidopsis thaliana*, BCAA, carbon stress, carbonic anhydrase, mitochondria

## Abstract

- Carbonic anhydrases (CAs) are ubiquitous metalloenzymes that catalyze the reversible hydration of CO_2_, enabling fundamental processes in organisms across all domains of life. Among all CAs, the role of mitochondrial βCA remains poorly understood.
- Here, we identify a mitochondrial βCA, βCA6, as a key regulator of branched‐chain amino acid (BCAA) catabolism and metabolic flexibility during carbon starvation in *Arabidopsis thaliana*.
- Loss of βCA6 triggers hypersensitivity to prolonged darkness, marked by accelerated Chl degradation, early senescence, impaired BCAA degradation, and disrupted carbon‐nitrogen remobilization. Transcriptomic and metabolic profiling revealed elevated expression of BCAA catabolic enzymes, as well as BCAA accumulation and reduced glutamate levels, indicating defective carbon‐nitrogen remobilization. *βca6* loss‐of‐function mutants exhibited a striking hypersensitivity to exogenous BCAAs, supporting a central role of βCA6 in BCAA homeostasis.
- These findings uncover a previously unrecognized function for mitochondrial CA in maintaining energy balance under dark stress. Given the evolutionary conservation of mitochondria and BCAA metabolism, our work highlights a broadly relevant mechanism by which eukaryotes integrate core metabolic pathways with environmental adaptation.

## Introduction

Metabolic flexibility, the ability to adjust substrate utilization in response to changing physiological conditions, is essential for energy homeostasis in eukaryotes (Goodpaster & Sparks, 2017; Galgani *et al*., 2022). In land plants, this adaptability is especially critical due to their photoautotrophic lifestyle and susceptibility to carbon limitation under stress or darkness. Central to this flexibility is the respiration network, which integrates diverse catabolic pathways to maintain ATP production and energy homeostasis under varying environmental conditions. This metabolic pathway flexibility is key to plant respiratory performance under fluctuating environmental conditions (O'Leary *et al*., 2019).

Branched‐chain amino acid (BCAA) catabolism has emerged as a key alternative pathway during carbon starvation in plants and metazoans (Wagenmakers *et al*., 1984; Araujo *et al*., 2011). Disruption of this pathway causes hypersensitivity to prolonged darkness, rapid degradation of cellular proteins, and early senescence in plants (Ishizaki *et al*., 2005, 2006; Araújo *et al*., 2010). The role of amino acids, including BCAAs, as alternative respiratory substrates is also relevant under moderate stress conditions, such as drought and salinity (Shim *et al*., 2023; Sun *et al*., 2024), while highlighting the importance of precise homeostatic control (Zhu & Galili, 2003; Angelovici *et al*., 2009; Cao *et al*., 2019).

Amino acid biosynthesis occurs predominantly in chloroplasts, while catabolism takes place in mitochondria and the cytosol (Kirk & Leech, 1972; Hildebrandt *et al*., 2015; Fortunato *et al*., 2023). During senescence, amino acid breakdown becomes increasingly important as chloroplasts degrade early, whereas mitochondria remain metabolically active until late stages (Avila‐Ospina *et al*., 2014). In mitochondria, BCAAs are degraded via a catabolic pathway, in which the key enzymes BCAT2, IVDH1, MCCA1, and ETFQO facilitate their integration into mitochondrial energy metabolism (Peng *et al*., 2015). BCAT2 catalyzes the first committed reaction of BCAA degradation by transaminating leucine, valine, and isoleucine into their corresponding α‐ketoacids (Angelovici *et al*., 2013). IVDH1 and MCCA1 function downstream as core dehydrogenase and carboxylase components that convert these intermediates into acyl‐CoA derivatives feeding into the tricarboxylic acid (TCA) cycle (Gu *et al*., 2010; Ding *et al*., 2012). ETFQO encodes the electron‐transfer flavoprotein : ubiquinone oxidoreductase, a pivotal electron‐accepting enzyme that links BCAA‐derived acyl‐CoA oxidation to mitochondrial respiration (Ishizaki *et al*., 2005). The biotin‐dependent enzyme 3‐methylcrotonyl‐CoA carboxylase (MCCase) catalyzes a key step that requires bicarbonate (HCO_3_^−^) as a substrate (Gallardo *et al*., 2001; Nikolau *et al*., 2003; Tong, 2013; Avila‐Ospina *et al*., 2014). This dependence on HCO_3_^−^ suggests a regulatory point linking carbon metabolism to amino acid catabolism (Anderson *et al*., 1998).

Carbonic anhydrases (CAs) are ubiquitous enzymes that catalyze the reversible hydration of CO_2_, thereby regulating cellular HCO_3_^−^ levels (DiMario *et al*., 2017). The *A. thaliana* genome contains 17 genes that encode α, β, and γ CA isoforms, including two γ‐like CAs. In *A. thaliana*, the βCAs are AtβCA1‐6. AtβCA1 and AtβCA5 are localized to the chloroplast, AtβCA2 and AtβCA3 are found in the cytosol, AtβCA4 is localized to the plasma membrane, and AtβCA6 is in the mitochondria (DiMario *et al*., 2017; Weerasooriya *et al*., 2022; Sharma *et al*., 2023). While plastidial β‐class CAs have well‐characterized roles in CO_2_/HCO_3_^−^ conversion (Raven, 2001; Hines *et al*., 2021), the function of mitochondrial βCAs remains unexplored.

In this study, we identify βCA6 as a mitochondrial βCA that plays a critical role in BCAA catabolism and metabolic adaptation to carbon starvation in *A. thaliana*. Through genetic, biochemical, and metabolomic analyses, we show that βCA6 activity is critical for maintaining BCAA homeostasis and supporting energy metabolism under stress. Our findings reveal a previously unrecognized role for mitochondrial CAs in coordinating amino acid degradation with energy balance, offering new insights into conserved mechanisms of metabolic flexibility across eukaryotes.

## Materials and Methods

### Plant material and growth conditions

*Arabidopsis thaliana* (L.) Heynh. Col‐0 ecotype was used as wild‐type (WT), and seeds of *βca6‐1* and *βca6‐2* mutant plants, corresponding to SALK_044658C (*βca6‐1*) and SALK_146554C (*βca6‐2*) (Col‐0 background), respectively, were obtained from the Arabidopsis Biological Resource Center (http://abrc.osu.edu). All seeds were surface‐sterilized by incubation with 25% (v/v) bleach, washed three times with sterile distilled water, and then plated on ½Murashige and Skoog (½MS) and 1/10th MS medium (pH 5.7), solidified with 0.8% (w/v) agar. The seeds were kept at 4°C for 3 d and germinated in the chamber under a photon flux density of 120 μmol m^−2^ s^−1^ and a 16 h : 8 h, light : dark photoperiod at 21°C. If needed, the seedlings were transferred from MS medium to soil 10 d after germination. The samples were harvested at midday and used for various metabolic and physiological measurements in the following experiments. Stable transformants were generated via a floral dip protocol (Clough & Bent, 1998). For the BCAA and glutamate supplementation assay, seedlings were grown on ½MS media without sucrose, supplemented with three different concentrations of BCAA (1, 1.5, and 2 mM) and 15 mM glutamate (Glu), along with the combination of both.

### Plasmid construction

The cDNA of βCA6 was PCR amplified from WT cDNA (leaf tissue) using primers listed in Supporting Information Table S1. The PCR products were cloned into Gateway‐compatible destination vector pEarlyGate 101 (Earley *et al*., 2006) for confocal microscopy analyses to generate the 35S::βCA6‐YFP constructs.

### T‐DNA mutant isolation and characterization

T‐DNA insertions were confirmed at the DNA level using PCR primers listed in Table S1. The expression of the *βCA6* gene was analyzed using reverse transcription‐polymerase chain reaction (RT‐PCR). RNA was extracted, and cDNA was synthesized from 1 μg of RNA as described previously (Pu *et al*., 2019). PCR was subsequently performed using the cDNA. *Ubiquitin 10* (*UBQ10*) was used for control purposes. The PCR consisted of 35 cycles of 95°C for 30 s, 50°C for 30 s, and 72°C for 1 min, followed by a 5 min 72°C extension.

### Subcellular localization analyses by confocal microscopy

Leaf samples were examined using a laser‐scanning confocal microscope (Nikon A1RS; Nikon Instruments, Tokyo, Japan). YFP fluorescence was detected using an excitation wavelength of 514 nm, with a bandpass filter having an emission range of 570–620 nm. For the detection of mitochondria, small leaf fragments (1 mm^2^) were immersed in a MitoTracker Red dye solution (CM‐H2XROS; Molecular Probes, Invitrogen) for 30 min and subsequently rinsed with water before observation. Excitation was carried out at 561 nm, and fluorescence was collected within the range of 590–610 nm. Images were analyzed using ImageJ software (Schindelin *et al*., 2012).

### Quantitative real‐time PCR analysis

Total RNA and cDNA were prepared from shoots and roots of WT and mutants as described above. qRT‐PCR was used to quantify the levels of mRNA‐encoding genes of *At1g10070* (*Branched‐Chain Amino Acid Transaminase 2, BCAT2*), *At3g45300* (*Isovaleryl‐CoA‐Dehydrogenase, IVD1*), *At2g43400* (*Electron‐Transfer Flavoprotein: Qbiquinone Oxidoreductase, ETFQO*), *At1g03090* (*Methylcrotonyl‐CoA Carboxylase Alpha Chain, MCCA1*), *At5g18170* (*Glutamate dehydrogenase, GDH1*), and *AT5G45890* (*Senescence‐Associated Gene 12, SAG12*). The primers used are listed in Table S1.

### Chl fluorescence measurements

The photochemical responses at PSII were obtained using 21‐d‐old WT and *βca6* mutants, grown at 21°C under white light (120 μmol m^−2^ s^−1^) with a long‐day photoperiod (16 h : 8 h, light : dark). Chl fluorescence was measured as previously described (Cruz *et al*., 2016). A 21‐d‐old plant was kept in a Dynamic Environmental Photosynthetic Imager (DEPI) chamber in the dark, and Chl fluorescence was estimated by image capture before, during, and after the application of saturating actinic illumination (120 μmol m^−2^ s^−1^) every day. *F*_v_ */F*_m_ was calculated using the image sequences (Baker & Oxborough, 2004). Data analysis was performed using software developed in‐house based on the open‐source software resources ImageJ (Schindelin *et al*., 2012).

### Chl estimation

Chl concentrations were assessed in both control and dark‐treated WT and *βca6* mutants. A total of 10 mg of fresh tissue was placed in 2 ml microcentrifuge tubes (MCT) containing 1 ml of dimethyl sulfoxide (DMSO). The MCTs were then incubated in a water bath at 65°C for 1 h. After cooling at room temperature for 30 min, the extracts were filtered, and absorption was measured at wavelengths of 663 and 645 nm. Chl levels were calculated according to previously established methods (Richardson *et al*., 2002).

### Dark respiration

Leaf gas exchange was assessed using a LI‐6400XT portable gas exchange system (LI‐COR, Lincoln, NE, USA). The analysis was conducted on 3‐wk‐old plants cultivated under long‐day conditions (16 h : 8 h, light : darkness at a temperature of 21°C). The leaf temperature was maintained at 21°C, reflecting the ambient conditions. We measured the respiration rate by quantifying the CO_2_ release under dark conditions following full dark adaptation. This was accomplished using an auto‐log program, which recorded measurements every 5 min over a 30‐min period.

### Determination of metabolite and amino acid levels by gas chromatography mass spectrometry

Metabolite analysis was conducted via gas chromatography mass spectrometry (GC–MS) to evaluate amino acids, organic acids, and sugars, employing an Agilent 7890 GC system coupled with an Agilent 5975C inert XL Mass Selective Detector (Agilent, Santa Clara, CA, USA). A total of 10 mg of 10‐d‐old WT and *βca6* mutant seedlings were cultivated on ½MS medium. Seedlings from both control and dark‐treated groups were harvested and immediately frozen in liquid nitrogen, followed by storage at −70°C until analysis. For extraction, the seedling powder was combined with a chloroform–methanol mixture (3 : 7) and incubated with 1 ml of this mixture at −20°C for 2 h, with occasional shaking. To facilitate quantification, an internal standard, specifically 50 μl of a 2 mg ml^−1^ ribitol solution, was added. Subsequently, 300 μl of Milli‐Q water (Merck KGaA, Darmstadt, Germany) was introduced to the sample and vortexed for 10–20 s to ensure thorough mixing. The mixture was then centrifuged at 2200 ***g*** at 4°C for 10 min. Aliquots of the methanol/water supernatant were freeze‐dried under vacuum for 6–16 h. Following this, the dried residue was dissolved and derivatized for 15 min at 60°C in 50 μl of 20 mg ml^−1^ methoxyamine hydrochloride in pyridine, followed by a 1‐h treatment with 50 μl of N‐methyl‐N‐(trimethylsilyl)trifluoroacetamide at the same temperature. An aliquot of the derivative was then injected into the GC–MS system. Signals were normalized to the internal standard (ribitol), allowing for the relative quantification of metabolites.

### Statistical analysis

For comparisons between two groups, a two‐tailed Student's *t*‐test was conducted. In time‐course experiments where the identical genotypes were measured repeatedly over multiple days (specifically *F*_v_/*F*_m_ measurements under prolonged dark treatment), a repeated‐measures ANOVA was applied, followed by Tukey's *post hoc* test to evaluate the differences between genotypes and time points using GraphPad Prism. Data are presented as mean ± SEM, and statistical significance was defined as ***, *P* < 0.001; **, *P* < 0.01; *, *P* < 0.05. Sample sizes (*n*) for each experiment are indicated in the figure legends.

## Results

### βCA6 is a mitochondrial enzyme that is induced by carbon starvation and is necessary to survive energy depletion

**Fig. 1:**
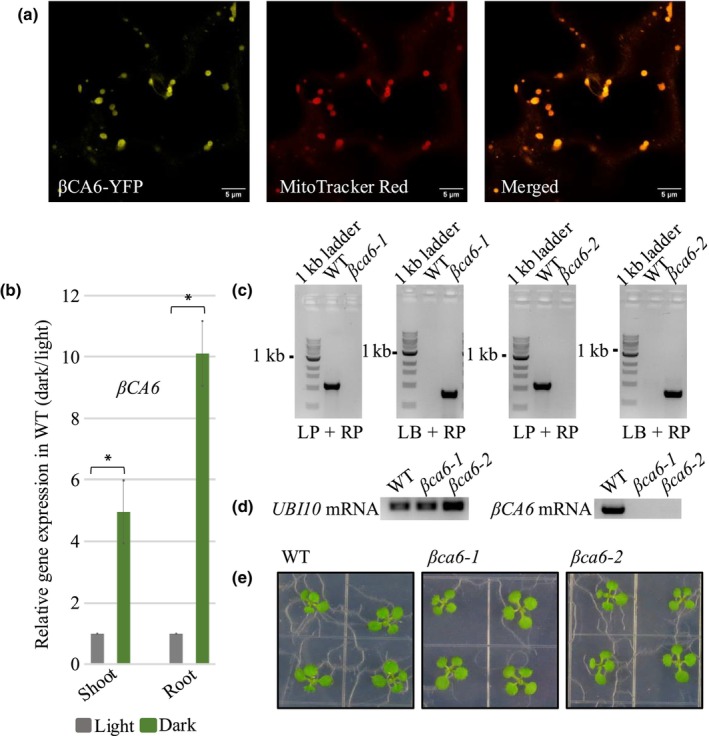
Characterization of βCA6 subcellular localization, *βCA6* expression, and *βCA6* KO mutant in *Arabidopsis* *thaliana*. (a) βCA6 is localized to the mitochondria as shown by colocalization with the mitochondrial marker MitoTracker Red (Invitrogen). Bar, 5 μm. (b) qRT‐PCR results of *βCA6* expression in shoots and roots of wild‐type (WT) upon 10 d of dark treatment. Relative expression levels are shown after normalization to *18S* and compared with the WT gene under light conditions (represented as onefold). Data are given as means ± SE (*n* = 3). An asterisk indicates a significant difference from the wild‐type, determined by the Student's *t*‐test (*, *P* < 0.05). (c) PCR‐based genotyping of two independent T‐DNA insertions in *βCA6* (SALK_044658C and SALK_146554C) shows an absence of transcript, unlike in WT. LP/RP refers to WT gene‐specific PCR for genes; LP/LB and RP/LB refer to T‐DNA insertion‐specific PCR for *βCA6* genes. LB is the T‐DNA left border primer. Gene‐specific primers and LB are shown in Table S1. (d) Characterization of the steady‐state mRNA levels of *βCA6* genes by RT‐PCR. *Ubiquitin 10* (*UBI10*) was used as the control, and gene‐specific primers are listed in Supporting Information Table S1. (e) Phenotypes of 10‐d‐old seedlings of βCA6 mutants, showing no apparent phenotype compared to the wild‐type (Col‐0).

To assess the subcellular localization of βCA6 (AGI: *At1g58180*) in the context of our experimental system, we generated a C‐terminal YFP fusion of the full‐length cDNA and expressed it in stable *A. thaliana* transgenic lines. Confocal microscopy analyses of 10‐d‐old seedlings revealed clear colocalization of βCA6‐YFP with MitoTracker (Invitrogen), a mitochondria‐specific dye (Fabre *et al*., 2007) (Fig. 1a), consistent with a prior report of its mitochondrial localization (Fabre *et al*., 2007). This validation within our study system reinforces the relevance of βCA6's mitochondrial targeting for subsequent functional analyses. We next examined *βCA6* mRNA levels and observed a significant increase under prolonged darkness in both shoots (5‐fold) and roots (10‐fold), which induces energy depletion (Gary *et al*., 2003) (Fig. 1b), in agreement with earlier findings (Wang *et al*., 2014), suggesting a potential role during metabolic stress adaptation under dark stress. To test this, we selected and characterized two independent T‐DNA insertion lines of *βCA6* from the SALK collection (SALK_044658C, *βca6‐1*, and SALK_146554C, *βca6*‐2), both carrying insertions within exons. Genomic PCR confirmed the insertions (Table S1; Fig. 1c), and RT‐PCR showed the absence of full‐length transcripts in homozygous lines (Fig. 1d). When grown under standard long‐day conditions (LD conditions; 16 h : 8 h, light : dark; 120 μmol m^−2^ s^−1^ PPFD; 21°C), the *βca6* lines exhibited no visible phenotypic differences compared to WT plants during the vegetative stage, except the increased transcript level of a senescence‐associated gene, that is *SAG12* (Figs 1e, S1a). To further evaluate whether nutrient limitation modifies the phenotypic response of the *βca6* lines, we grew WT and *βca6* seedlings on 1/10th strength MS medium, a highly diluted nutrient condition compared to ½MS used in our primary assays. Under light conditions, both WT and *βca6* lines displayed normal growth with no visible morphological differences (Fig. S2a,b).

**Fig. 2:**
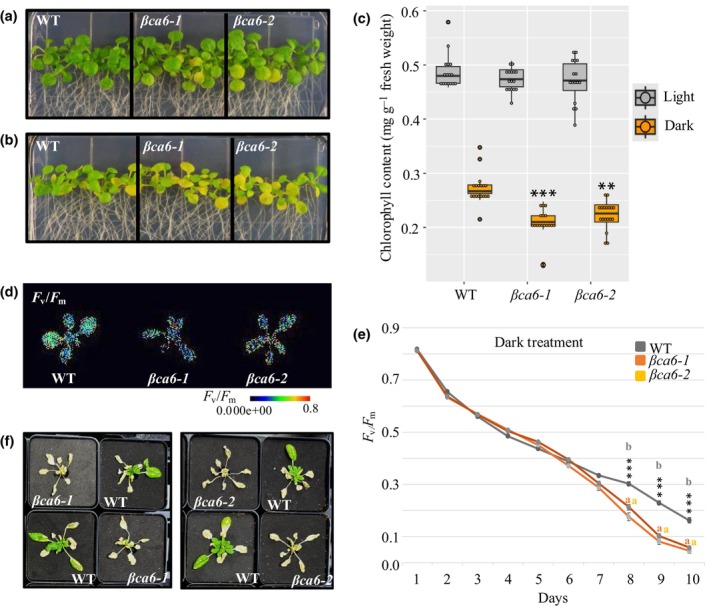
*βCA6* mutants exhibit enhanced sensitivity to extended dark in *Arabidopsis* *thaliana*. (a) Phenotypes of 20‐d‐old wild‐type (WT), *βca6‐1*, and *βca6‐2* under regular light regime on half MS media. (b) Photographs of 10‐d‐old seedlings of WT, *βca6‐1*, and *βca6‐2* after 10 d of prolonged darkness. The experiments were conducted at least three times, yielding consistent results, and representative data are presented. (c) Chl content control and dark‐treated WT, *βca6‐1*, and *βca6‐2*. Data are given as means ± SE (*n* = 3). An asterisk indicates a significant difference from the wild‐type, determined by the Student's *t*‐test (***, *P* < 0.001; **, *P* < 0.01). Boxplot display the first and third quartiles (the box), median (the horizontal line), and the minimum and maximum values (the whiskers). Outliers are plotted individually if they are more than 1.5 times the interquartile range from the hinge. (d) Photograph of 21‐d‐old wild‐type, *βca6‐1*, and *βca6‐2* plants after 10 d of dark treatment in DEPI chamber showing maximum photochemical efficiency of photosystem II (PSII) (*F*_v_/*F*_m_). (e) Analysis of the *F*_v_/*F*_m_ to quantify the kinetics of leaf senescence. (f) The phenotype of WT and *βca6* mutant plants after a 3‐d recovery period following 10 d of dark treatment in the DEPI chamber. Values are means ± SE of three biological replicates (plants = 10 per replicate). An asterisk indicates a significant difference from the wild‐type, determined by the repeated‐measures ANOVA, followed by Tukey's *post hoc*. Letters indicate groups by statistical significance (***, *P* < 0.001).

Next, to investigate the role of βCA6 in dark stress responses, we transferred 10‐d‐old *βca6* seedlings, grown under LD conditions, to continuous darkness. Within 10 d, all mutant lines exhibited pronounced chlorosis, a hallmark of leaf senescence, while the WT remained green and healthy (Fig. 2a,b). To further characterize this accelerated senescence, we assessed two standard chloroplast function and viability indicators: Chl content and photochemical efficiency of photosystem II, measured as maximum variable fluorescence/maximum fluorescence yield (*F*_v_ */F*_m_) (Oh *et al*., 1996). Under prolonged darkness, Chl levels declined significantly faster in *βca6* compared to WT (Fig. 2c). After 10 d of dark treatment, plants grown on 1/10th MS media displayed phenotypes comparable to those on ½MS media. Importantly, nutrient dilution did not introduce additional phenotypic differences or alter the characteristic dark‐sensitive phenotype of *βca6*. These results indicate that the interactions between βCA6 and carbon‐starvation pathways are not strongly influenced by bulk nutrient availability within the tested parameters (Fig. S2c,d).

We next monitored photochemical efficiency over a 10‐d period by transferring 3‐wk‐old *βca6* and WT plants into a DEPI chamber, an advanced imaging system designed to measure and visualize photosynthetic performance in plants under changing environmental conditions (Cruz *et al*., 2016). For this experiment, we used an extended dark stress treatment, where plants were kept in the dark in a DEPI chamber for 10 d, and *F*_v_ */F*_m_ was measured once daily. On day 0, *F*_v_ */F*_m_ values were comparable between genotypes (Fig. 2e). However, by day 7, *βca6* lines exhibited a marked decline in *F*_v_ */F*_m_, which was significantly lower than WT, indicating a loss of photosystem II function (Fig. 2d,e). Plants were returned to a standard long day (LD: 16 h : 8 h, light : dark) cycle for 3 d to evaluate recovery after dark stress. All WT plants resumed growth, whereas only a few of the *βca6* mutants recovered; the remainder showed irreversible senescence, with desiccated and yellowed leaves (Figs 2f, S3). We also checked the *SAG12* transcript level. We found that its expression increased strongly under dark treatment, and this response was markedly different between WT and *βca6* lines. Following 10 d of dark treatment, *SAG12* transcript level increased substantially in WT but rose even higher in the *βca6* lines, indicating an enhanced senescence response (Fig. S4a).

These findings demonstrate that βCA6 is essential for maintaining photosynthetic capacity and delaying senescence during prolonged darkness, highlighting its critical role in dark‐induced stress tolerance.

### A *βCA6* mutation induces the expression of BCAA catabolic genes

**Fig. 3:**
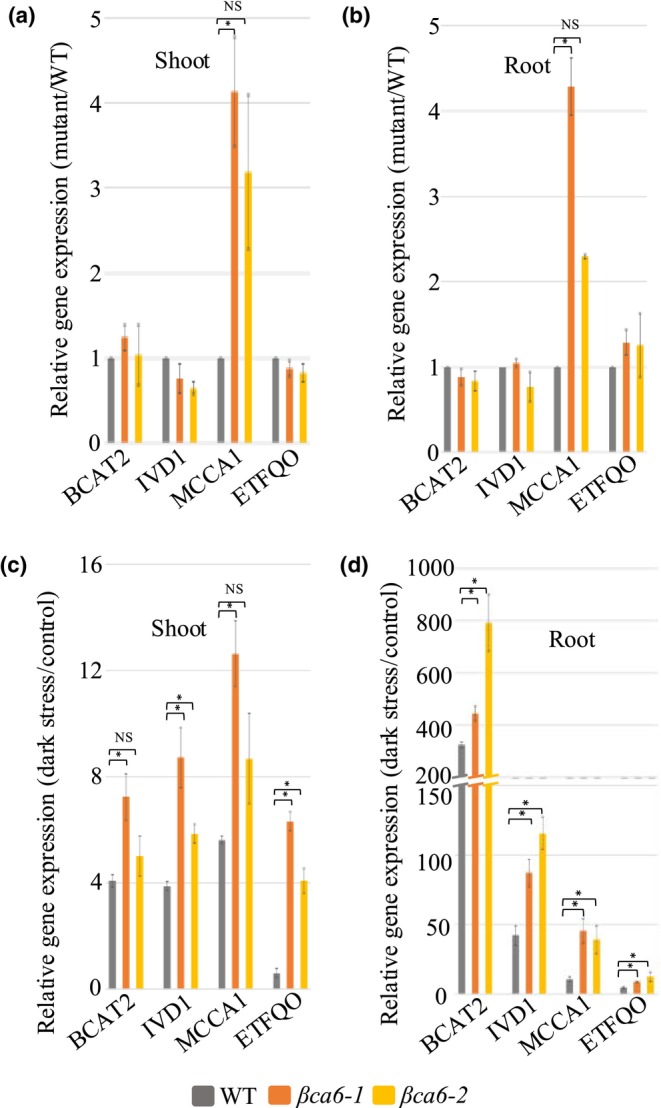
Gene expression analysis of BCAA catabolic genes in *βCA6* mutants in *Arabidopsis* *thaliana*. (a, b) Quantitative real‐time PCR results of BCAA catabolic genes in shoots and roots of the *βca6‐1* and *βca6‐2* under control conditions compared to wild‐type (WT), respectively. (c, d) Quantitative real‐time PCR results of BCAA catabolic genes in shoots and roots of the WT, *βca6‐1*, and *βca6‐2* under dark conditions compared to the control condition, respectively. Relative expression levels are reported after normalization to the *18S* expression level. Data are given as means ± SE (*n* = 3). An asterisk indicates a significant difference from the WT, determined by the Student's *t*‐test (*, *P* < 0.05).

Given the established role of BCAA catabolism in supporting energy production during carbon limitation (Peng *et al*., 2015; Bo & Fujii, 2025), we next examined the mRNA level of key BCAA catabolic genes in WT and *βca6* lines grown under standard LD conditions and after 10 d of continuous darkness. We focused on the transcript level of genes involved in early and downstream steps of BCAA degradation, including *At1g10070* (*Branched‐Chain Amino Acid Transaminase 2*, *BCAT2*), *At3g45300* (*Isovaleryl‐CoA‐Dehydrogenase, IVD1*), *At2g43400* (*Electron‐Transfer Flavoprotein: Q biquinone Oxidoreductase, ETFQO*), and *At1g03090* (*Methylcrotonyl‐CoA Carboxylase Alpha Chain*, *MCCA1*). Notably, *BCAT2*, *IVD1*, and *MCCA1* transcript levels rapidly increase upon imposition of darkness and are inhibited by sucrose (Peng *et al*., 2015). Under control LD conditions, we found that the *βca6* lines exhibited an increased expression of *MCCA1* compared to WT (i.e. fourfold and twofold higher, respectively) in both shoots and roots (Fig. 3a,b), indicating transcriptional increase of BCAA catabolic genes even in the absence of stress. After 10 d in darkness, WT plants showed a significant increase in BCAA‐degrading gene transcript level, with *BCAT2* level increasing by over 270‐fold relative to controls. A similar trend was observed for *IVD1*, *MCCA1*, and *ETFQO*. Similarly, under dark conditions in the *βca6* lines, we observed a significant increase in *BCAT2*, *IVD1*, and *MCCA1* transcript levels relative to control LD conditions and WT (Fig. 3c,d). The disproportionate rise of *MCCA1* in the *βca6* lines in light and extended darkness suggests a buildup of BCAA catabolic intermediates upstream of this step, resulting in a metabolic bottleneck. Further, we examined whether nitrogen–carbon balancing pathways are altered in the *βca6* lines by measuring *GDH1* under light and dark conditions in WT and *βca6* lines (Figs S1b, S4b). *GDH1* transcript level increased in both WT and *βca6* lines during dark treatment, but the increase was substantially greater in the mutant, indicating elevated nitrogen remobilization and Glu turnover during carbon starvation (Fig. S4b).

These results support a model in which βCA6 activity contributes to the efficient catabolism of BCAA, particularly under energy‐deprived conditions such as prolonged darkness. Disruption of *βCA6* is likely to impair this process, contributing to the early senescence phenotype observed in the mutants.

### βCA6 is required for BCAA catabolism and metabolic homeostasis during dark stress

**Fig. 4:**
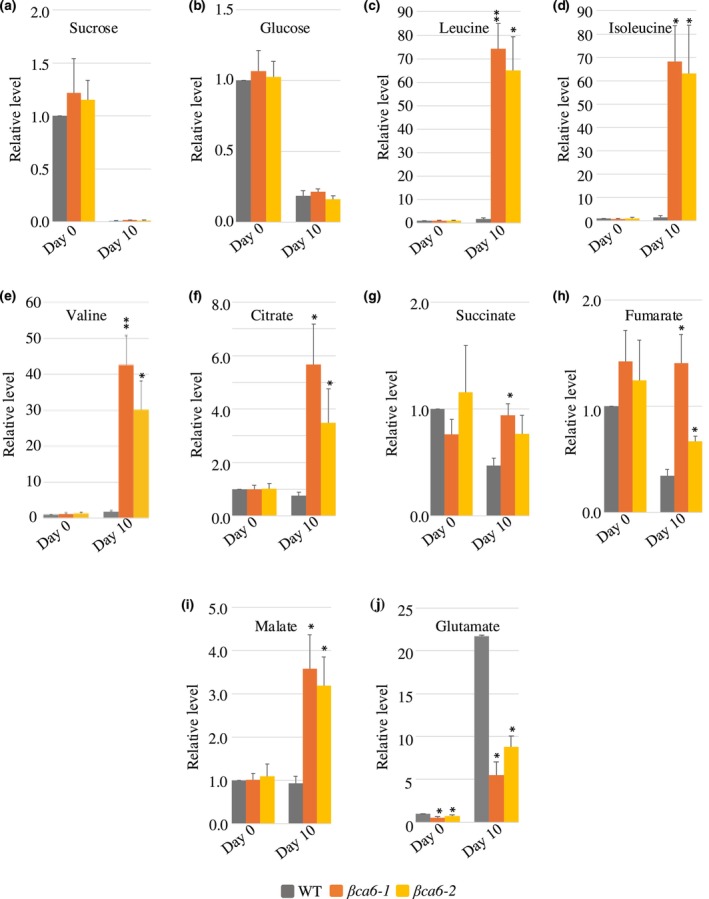
Metabolomics analysis of the *βca6* mutants in *Arabidopsis* *thaliana*. Relative levels of sugars, amino acids, and organic acids in wild‐type (WT), *βca6‐1*, and *βca6‐2* during extended dark conditions as measured by GC–MS. (a) Sucrose, (b) Glucose, (c) Leucine, (d) Isoleucine, (e) Valine, (f) Citrate, (g) Succinate, (h) Fumarate, (i) Malate, and (j) Glutamate. The *y*‐axis values represent metabolite levels relative to WT. Data were normalized to the mean response calculated for the untreated (0‐d dark‐treated) wild‐type. Values presented are means ± SE of determinations on five biological replicates. An asterisk indicates a significant difference from the wild‐type, determined by the Student's *t*‐test (**, *P* < 0.01; *, *P* < 0.05).

To determine whether the *βca6* lines exhibited altered metabolic profiles, we conducted GC–MS‐based metabolomic analyses of WT and *βca6* plants following 10 d of continuous darkness (Fig. 4). As expected, extended darkness led to a rapid depletion of sucrose and glucose in both WT and mutant lines (Fig. 4a,b). However, striking differences emerged in the accumulation of BCAAs: the levels of leucine, isoleucine, and valine were elevated more than 30‐fold in *βca6* mutants compared to WT (Fig. 4c–e), consistent with impaired BCAA degradation. This metabolic signature mirrors that of known BCAA catabolic mutants (Ishizaki *et al*., 2005; Peng *et al*., 2015), suggesting that *βCA6* plays a functional role in facilitating BCAA breakdown. During sucrose starvation, amino acid catabolism is typically activated to supply respiratory substrates to the TCA cycle (Contento *et al*., 2004). However, as in BCAA catabolic mutants, the accumulation of BCAAs in the *βca6* mutants indicates a blockage in this pathway. Notably, the *βca6* mutants also exhibited a marked reduction in glutamate (Glu) levels after dark treatment (Fig. 4j), a compensatory response previously associated with BCAA catabolic defects (Ishizaki *et al*., 2005). In such cases, enhanced Glu catabolism is thought to partially offset the deficit in respiratory substrates. Further profiling revealed significantly elevated levels of TCA cycle intermediates, including succinate, fumarate, and malate, in the *βca6* mutants under prolonged darkness (Fig. 4f–i).

Together, these results demonstrate that βCA6 is critical for maintaining metabolic homeostasis during energy deprivation, and its loss disrupts BCAA catabolism, leading to metabolite accumulation, Glu depletion, and reprogramming of central carbon metabolism.

### The *βCA6* mutation leads to enhanced dark respiration

**Fig. 5:**
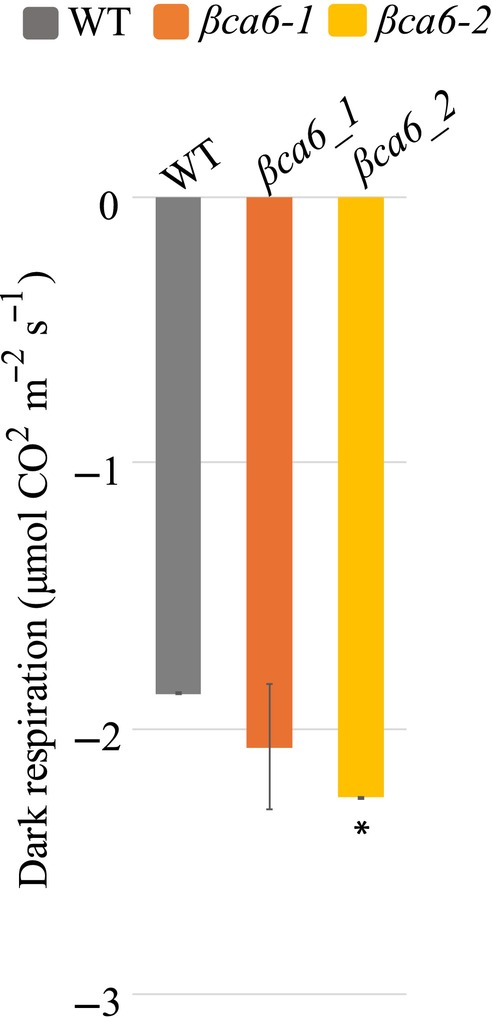
The respiration rate of *βca6* mutants in *Arabidopsis* *thaliana*. The respiration rate of 3‐wk‐old WT, *βca6‐1*, and *βca6‐2* during the dark. Values are means ± SE of three Plants. The experiment was repeated three times with similar results; a representative result is shown. An asterisk indicates a significant difference from the wild‐type, determined by the Student's *t*‐test (*, *P* < 0.05).

We hypothesized that βCA6 recaptures CO_2_ released in mitochondria and thereby regulates the mitochondrial HCO_3_^−^ pool for metabolic enzymes that require HCO_3_^−^ in mitochondria. To investigate this, we measured the CO_2_ release rate, or respiration, during the dark phase in both *βca6* mutants and WT plants. Our observations indicated that *βca6* mutants exhibited a higher rate of dark respiration compared to the WT (Fig. 5), which is consistent with a previous report (Jiang *et al*., 2014). Combined with the observation of a decreased respiration rate in *βCA6* overexpression lines (Jiang *et al*., 2014), our findings suggest that the elevated respiration rate in the *βca6* mutants may reflect a diminished capacity for the reversible hydration of CO_2_ in the absence of a functional *βCA6*.

### βCA6 loss leads to increased sensitivity to BCAA, which is reversed by Glu supplementation

**Fig. 6:**
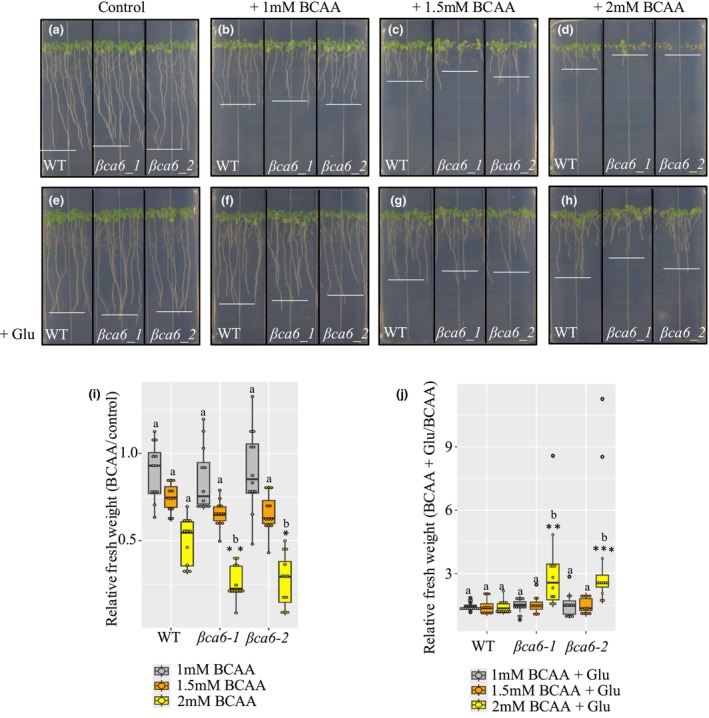
The loss of βCA6 in *Arabidopsis* causes growth arrest in conditions of high BCAA levels in the media. (a) Phenotype of wild‐type (WT), *βca6‐1*, and *βca6‐2* under control conditions. (b–d) Phenotype of WT, *βca6‐1*, and *βca6‐2* seedlings upon supplementation with 1, 1.5, and 2 mM BCAA, respectively. (e) Phenotype WT, *βca6‐1*, and *βca6‐2* upon glutamate supplementation. (f–h) Phenotype of WT, *βca6‐1*, and *βca6‐2* seedlings upon supplementation with 1, 1.5, 2 mM BCAA in addition to 15 mM glutamate (Glu), respectively. The white line represents the most frequent root length observed in the sample. (i, j) Relative fresh weight of WT, *βca6‐1*, and *βca6‐2* mutants grown on ½MS media supplemented with BCAA and BCAA plus Glu, respectively. Values presented are means ± SE of determinations on three biological replicates (seedlings = 30 per replicates). An asterisk indicates a significant difference from the control, determined by the two‐way ANOVA, followed by Tukey's *post hoc*. Letters indicate groups by statistical significance (***, *P* < 0.001; **, *P* < 0.01; *, *P* < 0.05). Boxplots display the first and third quartiles (the box), median (the horizontal line), and the minimum and maximum values (the whiskers). Outliers are plotted individually if they are more than 1.5 times the interquartile range from the hinge.

To assess whether βCA6 is required for effective BCAA catabolism under normal light : dark regime, we compared the growth of WT and the *βca6* mutants on media supplemented with increasing concentrations of BCAAs (1, 1.5, and 2 mM) (Fig. 6b–d). The *βca6* lines displayed severe, dose‐dependent growth inhibition. At 2 mM BCAA, 10‐d‐old *βca6* seedlings accumulated only 16% of the fresh weight observed in WT controls (Fig. 6d,i). Mutant phenotypes included stunted shoots, pale‐yellow cotyledons, and complete developmental arrest at the seedling stage (Fig. 6d). These results demonstrate that *βCA6* is essential for plant tolerance to elevated BCAA levels, supporting its role in BCAA catabolism under non‐stress conditions.

Since BCAA degradation generates ammonia, which is detoxified via Glu dehydrogenase and glutamine synthetase to form glutamine, Glu serves as a key nitrogen carrier in this process (Robinson *et al*., 1991; Sivanand & Vander Heiden, 2020; Mann *et al*., 2021). Therefore, to test whether impaired nitrogen assimilation contributes to the BCAA sensitivity of *βca6* mutants, we grew WT and mutant seedlings in 2 mM BCAA supplemented with 15 mM Glu (Fig. 6h,j). Remarkably, Glu addition fully rescued the growth inhibition phenotype of the mutants, suggesting that exogenous Glu mitigates ammonia toxicity, likely by facilitating transamination reactions that compensate for the disrupted BCAA catabolism.

In summary, *βca6* mutants are hypersensitive to elevated BCAA levels, and this sensitivity can be alleviated by Glu supplementation. These findings reinforce the role of βCA6 in maintaining amino acid and nitrogen homeostasis during BCAA turnover.

### βCA6 is required for transcriptional coordination of BCAA catabolism in response to metabolic inputs

**Fig. 7:**
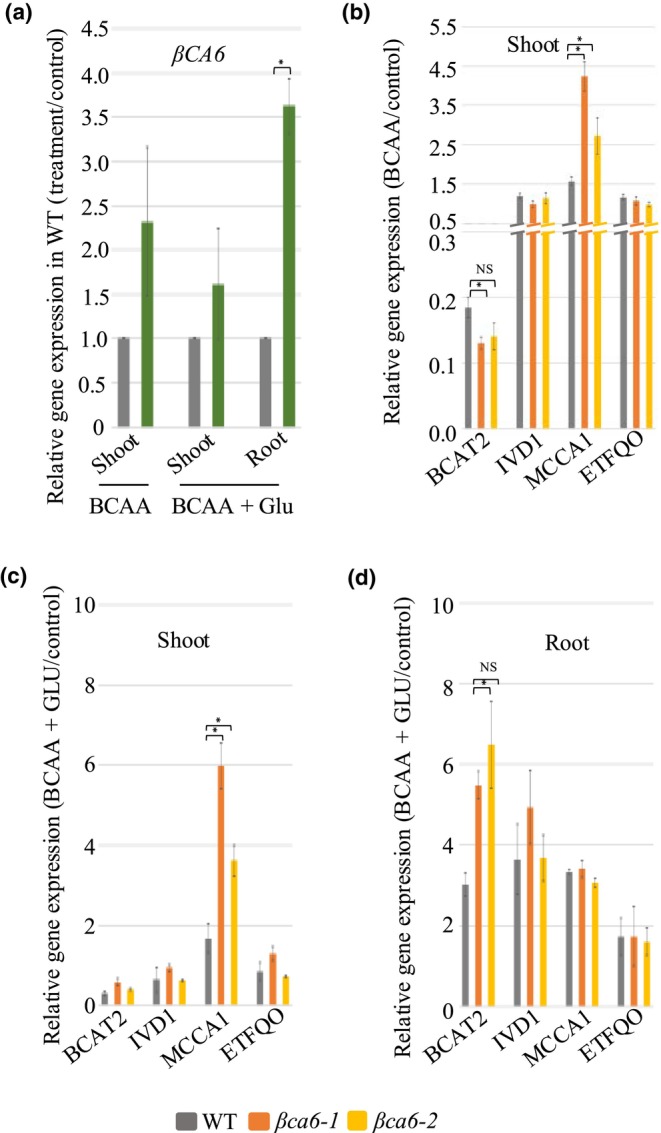
Gene expression of *βCA6* and BCAA catabolic genes upon BCAA supplementation in *Arabidopsis* *thaliana*. (a) Quantitative real‐time PCR results of βCA6 upon different treatments in shoots and roots. (b) Quantitative real‐time PCR results of BCAA catabolic genes in shoots of the wild‐type (WT), *βca6‐1*, and *βca6‐2* under BCAA treatment. (c, d) Quantitative real‐time PCR results of BCAA catabolic genes in shoots and roots of the WT, *βca6‐1*, and *βca6‐2* under BCAA + Glu treatment, respectively. Relative expression levels are given after being normalized to the expression level of *18S*. Data are given as means ± SE (*n* = 3). An asterisk indicates a significant difference from the wild‐type, determined by the Student's *t*‐test (*, *P* < 0.05).

Given the pronounced growth inhibition of the *βca6* mutants under exogenous BCAA supplementation and the rescue of this phenotype by Glu, we next examined how BCAA and Glu affect the expression of *βCA6* and key BCAA catabolic genes. In WT, BCAA supplementation increased *βCA6* transcript abundance. Notably, co‐supplementation with BCAAs and Glu led to an even greater increase in *βCA6* transcript level, up to 3.5‐fold (Fig. 7a), highlighting a strong transcriptional response to BCAA availability and further implicating *βCA6* in BCAA homeostasis. We also assessed the expression of key BCAA catabolic genes in WT and *βca6* lines under BCAA treatment alone and in combination with Glu. In WT, the *BCAT2* transcript decreased in shoots in response to BCAAs, indicating negative feedback inhibition (Fig. 7b). However, it increased to *c*. five to sixfold with BCAA + Glu co‐supplementation in roots (Fig. 7d), likely due to enhanced α‐ketoglutarate (α‐KG) availability supporting transamination reactions. By contrast, *βca6* mutants exhibited enhanced decrease in transcript level of *BCAT2* in response to BCAAs, likely due to enhanced accumulation of toxic BCAA intermediates (Fig. 7b). This was accompanied by a disproportionate increase in *MCCA1* transcript level, consistent with a metabolic bottleneck at the methylcrotonyl‐CoA carboxylase step. Significantly, Glu supplementation partially restored *MCCA1* transcript level to WT level in *βca6* in roots (Fig. 7d), suggesting that Glu mitigates transcriptional repression by replenishing α‐KG and buffering nitrogen imbalance. These results indicate that βCA6 is required for the coordinated transcriptional regulation of the BCAA degradation pathway. Although our transcript analysis revealed significant changes in the expression of *BCAT2*, *IVD1*, *MCCA1*, and *ETFQO*, we were unable to validate these patterns at the protein level due to the unavailability of specific antibodies. Therefore, these results should be interpreted with caution, as changes in transcript abundance may not always reflect changes in protein accumulation.

Collectively, our findings show that light‐grown WT plants dynamically regulate BCAA catabolic gene expression in response to substrate availability, whereas *βCA6* mutants exhibit impaired transcriptional flexibility due to metabolic gridlock, recoverable only through Glu supplementation, which bypasses the MCCA bottleneck and restores catabolic flux.

## Discussion

CAs are highly conserved enzymes that facilitate the reversible hydration of CO_2_ into HCO_3_^−^, a process essential for key metabolic functions, as uncatalyzed interconversion between CO_2_ and HCO_3_^−^ is slow when compared to the flux rates between these species, required in living cells (Badger & Price, 1994; Poschenrieder *et al*., 2018). Mitochondrial CA supplies HCO_3_^−^ for 3‐methylcrotonyl‐CoA carboxylase in the leucine degradation pathway, thus aiding in BCAA catabolism (Marwaha *et al*., 2021). Additionally, HCO_3_^−^ from mitochondrial CA may participate in pathways involving acetyl‐CoA carboxylase (ACCase) and pyruvate carboxylase, linking mitochondrial function to lipid biosynthesis and anaplerotic CO_2_ fixation (Marwaha *et al*., 2021). Despite the importance of HCO_3_^−^ in these mitochondrial pathways, the understanding of the roles of mitochondrial CA in plants remains limited. Our study addresses this gap by demonstrating that βCA6 is crucial for BCAA catabolism and metabolic flexibility under carbon stress conditions.

Although the functions of mitochondrial γCAs have been previously described in *A. thaliana* (Parisi *et al*., 2004; Fromm *et al*., 2016a,b), the roles of mitochondrial β‐class CAs have remained unclear. Here, we identify Arabidopsis βCA6 as a mitochondrial βCA that becomes critical to survival under carbon‐limiting conditions. Indeed, the lack of visible phenotypes in the *βca6* mutants under normal growth conditions indicates a dispensable role during photoautotrophic growth, with βCA6 becoming critical only during metabolic stress. However, under extended darkness, the absence of βCA6 led to rapid Chl loss, reduced photosystem II efficiency, and accelerated senescence, hallmarks of impaired metabolic flexibility. These phenotypes underscore the requirement for βCA6 in maintaining energy homeostasis when carbon availability is limited. In addition to confirming the subcellular localization in mitochondria (DiMario *et al*., 2017), we observed a marked induction of *βCA6* expression during prolonged darkness, consistent with an adaptive response to energy stress. This expression pattern parallels that of other mitochondrial enzymes involved in alternative respiratory pathways, suggesting coordinated regulation under carbohydrate starvation (Peng *et al*., 2015).

Experimentally induced sucrose starvation causes a marked decrease in expression of genes involved in carbohydrate breakdown, glycolysis, the TCA cycle, mitochondrial electron transport, and ATP synthesis (Buchanan‐Wollaston *et al*., 2005; Ishizaki *et al*., 2005). This is associated with a decrease in respiratory energy metabolism and increased expression of several genes involved in proteolysis, amino acid catabolism, and fatty acid degradation, suggesting the induction of salvage mechanisms that release amino acids and fatty acids from structural proteins and lipids as an alternative source of respiratory substrate to maintain viability of carbohydrate‐starved cells (Contento *et al*., 2004; Lin & Wu, 2004; Thimm *et al*., 2004; Buchanan‐Wollaston *et al*., 2005; Ishizaki *et al*., 2005). Our transcriptomic analyses revealed an increased expression of *βCA6* in dark‐adapted plants. Furthermore, we found a marked hypersensitivity of the *βca6* mutants to extended darkness, which suggests a role for βCA6 in utilizing an alternative source of respiratory substrates, such as those derived from proteolysis and/or lipid degradation. The observed accelerated Chl degradation and decrease in photosystem II efficiency indicate that the senescence is more rapid in the *βca6* mutants than in WT plants under extended dark conditions, which is bolstered by the highly pronounced expression of the senescence marker gene, *SAG12*. Moreover, a reduced recovery rate of the *βca6* mutants after dark treatment compared to WT further confirms that βCA6 is necessary for maintaining viability during prolonged carbon limitation, supporting a role for this enzyme in mitochondrial energy homeostasis.

Following a diel cycle and during prolonged darkness, BCAA catabolism genes are highly expressed during the night (Peng *et al*., 2015). Our transcriptomic analyses revealed a striking upregulation of BCAA catabolic genes in *βca6* mutants, particularly the two‐ to fourfold induction of *MCCA* under normal light conditions, indicating a feedback response to disrupted BCAA turnover. Despite this transcriptional response, BCAA accumulation was not detected under physiological growth conditions, suggesting that compensatory pathways partially buffer the metabolic defect in the presence of light. By contrast, during extended darkness, *βCA6* mutant plants robustly induce the BCAA catabolism pathway specifically in roots. Notably, *MCCA1*, which encodes the carboxylase responsible for converting 3‐methylcrotonyl‐CoA to 3‐methylglutaconyl‐CoA, was significantly upregulated in the mutants, suggesting a bottleneck at this step and implicating HCO_3_^−^ limitation as a contributing factor.

**Fig. 8:**
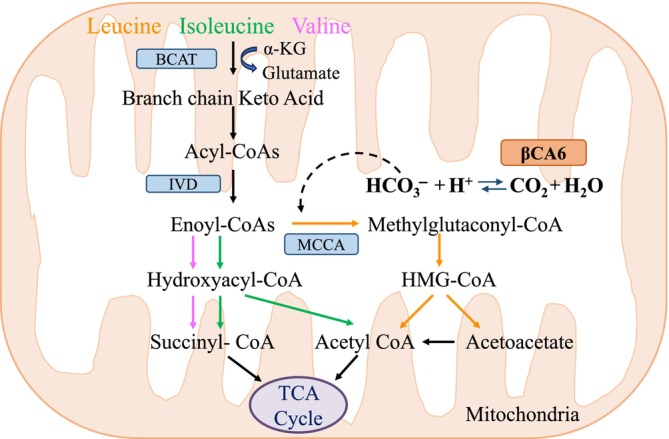
A simple model based on findings and published data regarding the role of mitochondrial‐localized βCA6 in *Arabidopsis* *thaliana* in regulating branched‐chain amino acid (BCAA) catabolism. βCA6 catalyzes the reversible hydration of CO_2_ and might supply bicarbonate to the methylcrotonyl‐CoA carboxylase (MCCase) during the conversion of methylglutaconyl‐CoA to 3‐hydroxy‐3‐methylglutaryl‐CoA (HMG‐CoA). A mutation in the βCA6 gene would affect leucine catabolism and inhibit overall BCAA catabolism. Solid lines represent established BCAA catabolic pathways. The dashed line indicate our proposed utilization of bicarbonate by MCCase.

Our findings support a model in which βCA6 catalyzes the reversible hydration of CO_2_ and may supply HCO_3_^−^ to MCCA, a critical enzyme in leucine catabolism (Fig. 8). The loss of βCA6 impairs this step, leading to the accumulation of BCAAs and intermediates, the depletion of Glu and glutamine pools, and metabolic gridlock. A similar pattern of BCAA accumulation and reduced Glu is observed in BCAA catabolic mutants (Ishizaki *et al*., 2005; Peng *et al*., 2015). The increased levels of TCA cycle intermediates (e.g. succinate, malate, and fumarate) may reflect both a compensatory upregulation of respiration and a secondary consequence of disrupted BCAA degradation, as for every molecule of leucine catabolism, there is a reduction of one molecule of the TCA cycle (Wagenmakers *et al*., 1990). The observed decrease in Glu and increased expression of *GDH1* upon dark treatment indicate that α‐KG is supplied to the TCA cycle. Glutamate acts as an essential anaplerotic precursor, and reduced levels indicate its consumption for running the TCA cycle. A similar phenomenon is observed in skeletal muscle at the onset of exercise; there is a dramatic drop in intramuscular glutamate levels while intermediates of the tricarboxylic acid (TCA) cycle rise *c*. fourfold (Bowtell & Bruce, 2002; Miyashita & Good, 2008). Moreover, α‐KG and Glu are required for both transamination and ammonia detoxification as well (Wagenmakers *et al*., 1990; Robinson *et al*., 1991; Sivanand & Vander Heiden, 2020; Mann *et al*., 2021). This is corroborated by the observation of an increase and a decrease in respiration rate in *βca6* mutant and overexpression, respectively (Jiang *et al*., 2014). We therefore propose that impaired metabolic flexibility in the *βCA6* mutants increases reliance on alternative catabolic pathways, resulting in a higher basal respiratory rate under dark‐induced carbon starvation. These metabolic disruptions were exacerbated when *βCA6* mutants were exposed to exogenous BCAAs, resulting in dose‐dependent growth inhibition even under normal light conditions. Supplementation with Glu partially rescued this phenotype, consistent with its role in buffering ammonia toxicity and supporting transamination reactions (Wagenmakers *et al*., 1990). Therefore, our results support that βCA6 is required not only during carbon starvation but also for maintaining BCAA homeostasis under standard conditions.

Importantly, parallels between *A. thaliana* βCA6 and human mitochondrial CAs (e.g. CA VA and CA VB) suggest deep evolutionary conservation of function. In humans, CA VB supplies HCO_3_^−^ to MCC during leucine degradation, and deficiencies lead to toxic BCAA accumulation and metabolic stress (Gallardo *et al*., 2001; van Karnebeek *et al*., 2014). Although structurally distinct, βCA6 belongs to the β‐class and human CAs to the α‐class; both enzymes converge on a shared biochemical role: enabling bicarbonate‐dependent carboxylation critical for amino acid catabolism. The similarity in metabolic phenotypes between βCA6‐deficient plants and CA VB‐deficient humans highlights a conserved mechanism by which mitochondrial CAs function as metabolic rheostats, integrating inorganic carbon balance with nitrogen metabolism.

Taken together, our results reveal a previously unrecognized function of mitochondrial CA in supporting amino acid catabolism and stress resilience in plants. More broadly, they point to an evolutionarily conserved strategy in eukaryotes to couple bicarbonate availability with metabolic flexibility. Understanding how mitochondrial CAs regulate these pathways offers new insights into plant stress responses and may inform therapeutic approaches for human disorders linked to organic acid metabolism.

## Competing interest

None declared.

## Author contributions

NS and FB designed research; NS performed research; NS, TDS and FB analyzed data; NS and FB wrote the paper.

## Disclaimer

The New Phytologist Foundation remains neutral with regard to jurisdictional claims in maps and in any institutional affiliations.

## Supporting information


**Fig. S1** Gene expression analysis of *SAG12* (*senescence‐associated gene 12*) and *GDH1* (*Glutamate Dehydrogenase 1*) genes.
**Fig. S2** Phenotypic analysis of the *βca6* mutant and WT on ½MS and 1/10th MS.
**Fig. S3** The phenotype of WT and *βca6* mutant plants after a 3‐d recovery period following 10 d of dark treatment in the DEPI chamber.
**Fig. S4** Gene expression analysis of *SAG12* (*senescence‐associated gene 12*) and *GDH1* (*Glutamate Dehydrogenase 1*) genes.
**Table S1** List of primers used in the study.
Please note: Wiley is not responsible for the content or functionality of any Supporting Information supplied by the authors. Any queries (other than missing material) should be directed to the *New Phytologist* Central Office.

## Data Availability

All the relevant data are provided in the main text (Figs 1, 2, 3, 4, 5, 6, 7) and its Supporting Information (Table S1; Figs S1, S4).
