# Supplementary material for: Mitochondrial β‐carbonic anhydrase is a conserved metabolic rheostat for branched‐chain amino acid catabolism and metabolic flexibility
Source: New Phytol. 2026 Mar 15;250(4):2415–27. doi: 10.1111/nph.71056 (PMC13103436; doi:10.1111/nph.71056)
Supplement: Supplementary file 1 — Fig. S1 Gene expression analysis of SAG12 (senescence‐associated gene 12) and GDH1 (Glutamate Dehydrogenase 1) genes. Fig. S2 Phenotypic analysis of the βca6 mutant and WT on ½MS and 1/10th MS. Fig. S3 The phenotype of WT and βca6 mutant plants after a 3‐d recovery period following 10 d of dark treatment in the DEPI chamber. Fig. S4 Gene expression analysis of SAG12 (senescence‐associated gene 12) and GDH1 (Glutamate Dehydrogenase 1) genes. Table S1 List of primers used in the study. Please note: Wiley is not responsible for the content or functionality of any Supporting Information supplied by the authors. Any queries (other than missing material) should be directed to the New Phytologist Central Office. [file NPH-250-2415-s001.pdf]

New Phytologist Supporting Information

Article title: Mitochondrial β-Carbonic Anhydrase Is a Conserved Metabolic Rheostat for Branched-Chain Amino Acid Catabolism and Metabolic Flexibility

Authors: Naveen Sharma, Thomas D. Sharkey, Federica Brandizzi

Article acceptance date: 9 February 2026

Table S1. List of primers used in the study.

	Primer Name	Primer 5' to 3'
**1**	AtβCA6-F	GGGGACAAGTTTGTACAAAAAAGCAGGCTATGGCGTTTACACTAGGTGG
**2**	AtβCA6-R	GGGGACCACTTTGTACAAGAAAGCTGGGTTACTCCATATCTCTCTGTCTGA
**3**	AtβCA6_qRT-F	AAATGTCGCCAATCTCGT
**4**	AtβCA6_qRT-R	ATGTTCTCAACCTGAAGAGTG
**5**	βca6-1_LP	TCTTAACTTAGCGGCTTTCCC
**6**	βca6-1_RP	GAAGCTGCAACAAATTGGATC
**7**	βca6-2_LP	TACCCTTGAATCTGCACATCC
**8**	βca6-2_RP	CTTGTGACCAGGCAAAGAGAC
**9**	BCAT2_qRT_F	TCACAAATTATGCGCCAGTT
**10**	BCAT2_qRT_R	CGAGATAAAGAACGTCTGAAAACC
**11**	MCCA1_qRT_F	AGAGACATGGGTGATAAAAGTGC
**12**	MCCA1_qRT_R	TATCCAGGCACGAGAGGAAC
**13**	IVD1_qRT_F	AATGGGAAAGTTGACCCAAAGGAC
**14**	IVD1_qRT_R	TAAAGCGACCTGCGTTGCTCTC
**15**	ETFQO1_qRT_F	TTGGCCATTAGTGCTATGGAACAC
**16**	ETFQO1_qRT_R	TCCCATGCTTGAGCGTGAAAGG
**17**	GDH1_qRT_F	TTGGTTGTGATCCTAGCAAGCTC
**18**	GDH1_qRT_R	AAGAATCCAAGCCATTGTCTGAGG
**19**	SAG12_qRT_F	TGCTTTGCCGGTTTCTGTTG
**20**	SAG12_qRT_R	AAAACGCCCAACAACATCCG

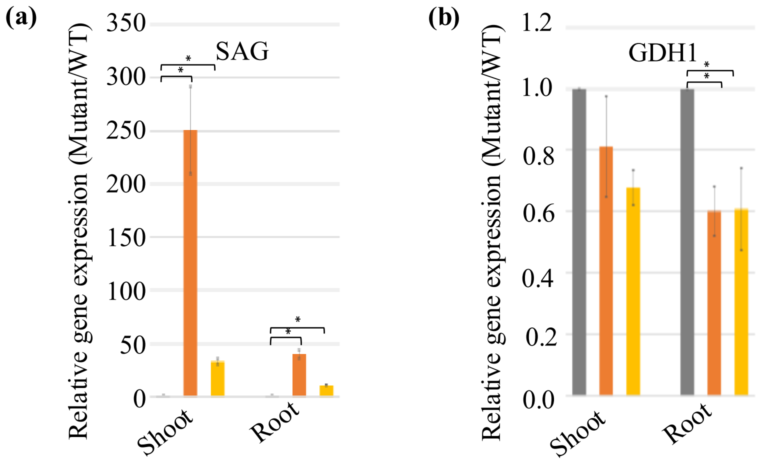

Fig. S1. Gene expression analysis of *SAG12* (senescence-associated gene 12) and *GDH1* (Glutamate Dehydrogenase1) genes.
(a, b). Quantitative real-time PCR results of *SAG12* and *GDH1* in shoots and roots of the *βca6-1* and *βca6-2* under control conditions compared to WT. Relative expression levels are reported after normalization to the *18S* expression level and compared with the control condition (represented as 1-fold). Data are given as means +_ SE (n = 3). An asterisk indicates a significant difference from the wild type, determined by the Student's t-test (***p < 0.001, **p < 0.01,*P < 0.05).

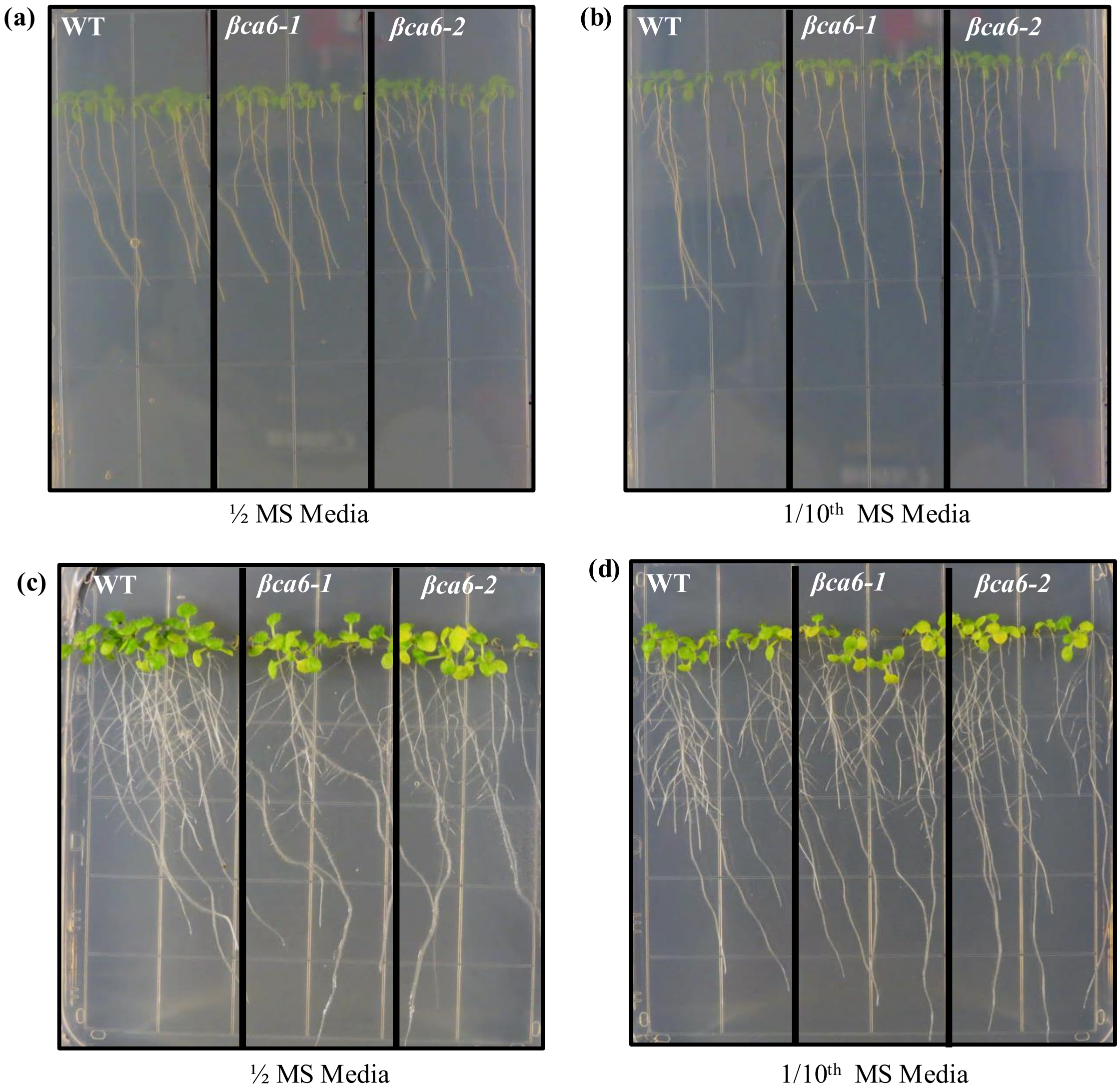

Fig. S2. Phenotypic analysis of the *βCA6* mutant and WT on ½ MS and 1/10th MS

(a), (b). Phenotype wild type (WT), *βca6-1*, and *βca6-2* under control conditions on ½ MS and 1/10th MS media, respectively. (c), (d). Phenotype of WT, *βca6-1,* and *βca6-2* seedlings upon 10 days of dark treatment on ½ MS and 1/10th MS media, respectively.

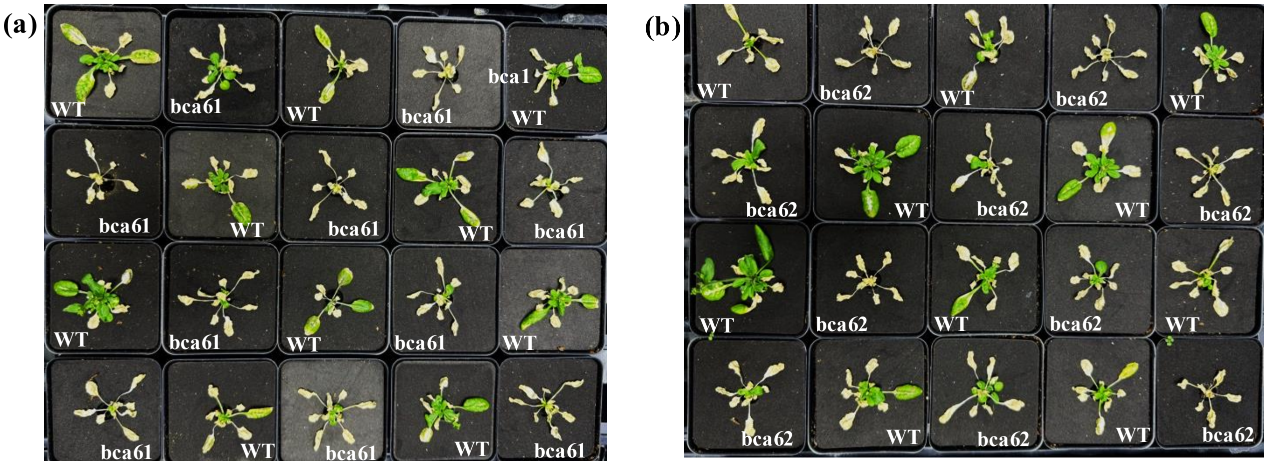

Fig. S3

The phenotype of WT and *βca6* mutant plants after a 3-day recovery period following 10 days of dark treatment in DEPI chamber

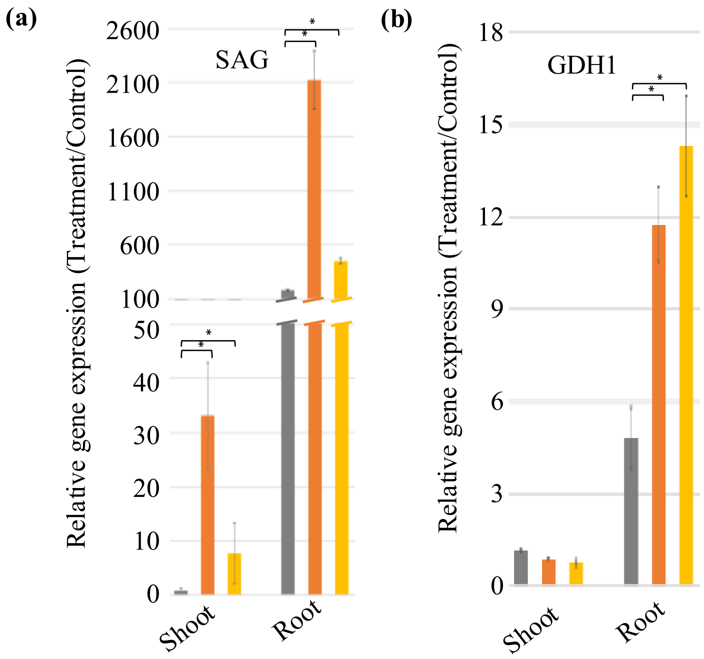

Fig. S4. Gene expression analysis of *SAG12* (senescence-associated gene 12) and *GDH1* (Glutamate Dehydrogenase1) genes.

(a, b). Quantitative real-time PCR results of *SAG12* and *GDH1* in shoots and roots of the *βca6-1* and *βca6-2* upon 10 days of dark treatment compared to WT. Relative expression levels are reported after normalization to the *18S* expression level and compared with the control condition (represented as 1-fold). Data are given as means +_ SE (n = 3). An asterisk indicates a significant difference from the wild type, determined by the Student's t-test (***p < 0.001, **p < 0.01,*P < 0.05).
